# Effectiveness of Smart Continence Care for People With Profound Intellectual and Multiple Disabilities: Cluster Randomized Trial

**DOI:** 10.2196/66389

**Published:** 2025-07-31

**Authors:** Vivette JC van Cooten, Marieke FM Gielissen, Wouter den Hollander, Ghislaine APG van Mastrigt, Odile Smeets, Inge MB Bongers, Brigitte Boon

**Affiliations:** 1 Academy Het Dorp, Research & Advisory on Technology in Long-term Care Arnhem The Netherlands; 2 Tranzo Tilburg School of Social and Behavioral Sciences Tilburg University Tilburg The Netherlands; 3 Trimbos Institute Netherlands Institute of Mental Health and Addiction Utrecht The Netherlands; 4 Department of Health Services Research Care and Public Health Research Institute (Research School CAPHRI) Maastricht University Maastricht The Netherlands; 5 Siza Center for Long-term Care for People with Disabilities Arnhem The Netherlands

**Keywords:** trial, profound intellectual and multiple disabilities, eHealth, electronic monitoring, incontinence, smart diaper, implementation, effect study, residential care, implementation

## Abstract

**Background:**

People with profound intellectual and multiple disabilities (PIMD) living in residential care facilities receive continence care at scheduled times. This can result in leakage or unnecessary incontinence material (IM) changes, negatively affecting individuals and their professional caregivers. Smart continence care (SCC), which notifies caregivers when IMs require changing, may resolve these issues and improve personalized care.

**Objective:**

We aimed to evaluate the effects of SCC in comparison with regular continence care. The primary outcome measure was the number of weekly leakages. Secondary outcomes were the number of weekly IM changes, the time spent on continence care, the quality of life of individuals with PIMD, and caregivers’ physical burden.

**Methods:**

A staggered-entry, open-label, cluster randomized trial was conducted in residential care facilities for people with disabilities in the Netherlands. Overall, 165 participants with PIMD and incontinence who were unable to indicate the need for IM changes were recruited, of whom 156 (94.5%) were included in the analyses. Six residential care facilities participated in the study and were randomized at a cluster level of 1:1. The intervention group (82/156, 52.6%) received SCC for 12 weeks. The waiting-list group (74/156, 47.4%) continued to receive regular continence care. Outcome measures were collected at weeks 0, 6, and 12 and were analyzed using generalized linear mixed models.

**Results:**

In intention-to-treat analyses comparing both groups, SCC appeared ineffective in reducing weekly leakages. An unexpected reduction in leakages for the waiting-list group compared to the intervention group was revealed (β coefficient=1.013, 95% CI 0.217-1.808; *P*=.01). SCC was effective in reducing the number of IM changes per week (β coefficient=−2.005, 95% CI −3.303 to −.707; *P*=.003). No significant reduction in the time spent on continence care (*P*=.84) was observed. There was no effect on quality of life or improvement in caregivers’ physical burden. Per-protocol and completer analyses did not alter our intention-to-treat conclusions. However, exploratory analyses revealed that the counterintuitive effect on leakage may be due to increased leakages at most locations within one residential care facility in the intervention group. The exclusion of this facility from the analyses revealed that the effect on leakage, which had previously favored the waiting-list group, was no longer significant. The reduction in the number of IM changes in the intervention group compared to the waiting-list group showed an increased effect size, from Cohen *d=*−0.34 to Cohen *d*=−0.45.

**Conclusions:**

SCC was ineffective in reducing the number of weekly leakages, but it reduced the number of weekly IM changes. We observed improvements in continence care in both groups. Increased attention to continence care, with or without technology, may lead to improved care outcomes. Additional research is required to identify the settings in which the implementation of SCC is most meaningful.

**Trial Registration:**

ClinicalTrials.gov NCT05481840; https://clinicaltrials.gov/ct2/show/NCT05481840

**International Registered Report Identifier (IRRID):**

RR2-10.2196/42555

## Introduction

### Background

Approximately 9000 persons with profound intellectual and multiple disabilities (PIMD) live in Dutch residential care facilities for people with disabilities [1,2]. Up to 96% of this group experience from some form of incontinence, although the estimates of prevalence vary between studies [3]. In general, people with PIMD have multiple physical disabilities and medical problems [4], and because of their severe cognitive disability, they are unable to inform their professional caregivers (hereafter referred to as caregivers) about the need to change incontinence materials (IMs). Therefore, most IMs are changed according to a fixed schedule [5]. However, scheduled IM changes may result in leakages when the material is oversaturated, causing urine or feces, or both, to leak outside the IM, or in unnecessary changes when the material is still dry. In case of a leakage, caregivers need to change not only the IM but also clothes and bedsheets, and occasionally shower the person. This is burdensome and affects the quality of life of individuals with PIMD. If leakages, as well as unnecessary IM changes, can be prevented, continence care may become more personalized and improve the quality of life of the person with PIMD along the way [5]. Moreover, more accurate IM changes could result in caregivers spending less time on the aforementioned incontinence-related activities, which may positively affect them, as caring for people with PIMD is physically demanding [5].

Smart continence care (SCC), which uses materials with integrated urine sensors, may resolve negative issues related to regular continence care (RCC), offering the possibility of on-demand and more personalized care. Various SCC technologies have appeared on the market, each with its own specifications. Different types and brands of smart continence products have been the subject of investigations globally [6-10]. These studies showed that SCC can improve incontinence care for persons in long-term care facilities. However, most studies on SCC have predominantly focused on its use in the care of vulnerable older adults, with small sample sizes (eg, n=35 [6] and n=31 [8]). A Canadian study on care for older people [7] had a larger sample size (n=101) and showed that using an SCC solution with 72-hour monitoring of the voiding pattern was helpful in constructing a personalized care plan. In the Netherlands, several small pilot studies on SCC conducted in nursing homes for the older adults have revealed positive results supporting its use [9,10].

Although most of these studies show some potential for improving continence care by reducing leakage [6,7,9,10], reducing the number of IM changes [9,10], and positively affecting patients and their caregivers [6-10], these results cannot be generalized to people with PIMD living in residential care facilities. People with PIMD can have different body postures because of their physical disabilities, which affects how IMs fit the person. Furthermore, they may experience different medical conditions that induce incontinence; for example, epilepsy, which is more common in this group, can lead to incontinence [11]. In addition, older people stay, on average, for shorter periods in residential care facilities [12] and show age-related decline. In contrast, people with disabilities in residential care facilities often live there for many years in more or less stable health conditions. Thus, it is important to study the effectiveness of SCC in residential care and specifically evaluate its use in people with PIMD.

Implementing SCC in organizations is complex as different teams and departments need to be involved and work routines must change. Care teams from day and night care, as well as day activity centers, need to collaborate and agree on how to integrate this new technology into their daily work routines. Changing IMs based on notifications generated by sensors requires letting go of fixed schedules and adjustments to work routines [5,13]. Moreover, the facility’s IT department is required to develop the technical infrastructure needed for the SCC technology and provide technical support.

Overall, the use of SCC seems promising for people with PIMD who experience incontinence within residential care. However, reliable evidence to confirm its effectiveness is lacking because SCC has not yet been comprehensively evaluated in a clinical trial setting for people with PIMD. Therefore, in this study, a cluster randomized trial was conducted to examine the effectiveness of SCC, as compared to RCC, for people with PIMD living in Dutch residential care facilities.

### Objectives

We aimed to evaluate whether SCC indeed results in a reduced number of leakages, a decreased number of IM changes, and an improved quality of life for people with PIMD, as well as a smaller incontinence care–related burden on their caregivers as compared to RCC.

## Methods

The detailed study protocol for this trial was previously published [13], which described the research design for the effectiveness study presented in this paper, as well as an economic evaluation. This study followed the CONSORT (Consolidated Standards of Reporting Trials) guidelines [14-18] (Multimedia Appendix 1).

### Study Design

A staggered-entry open-label cluster randomized trial was conducted in the Netherlands from September 2021 to April 2023 to compare SCC and RCC. The primary outcome was the number of weekly leakages at baseline (T0), week 6 (T1), and week 12 (T2). Secondary outcomes were the number of weekly IM changes and the amount of time spent on continence care (both measured at T0, T1, and T2), as well as the quality of life of individuals with PIMD and caregivers’ physical burden experienced during continence care (both measured at T0 and T2). A cluster design was chosen so that either implementation of the intervention or provision of regular care was similar throughout the teams, as the intervention impacted the caregivers’ work routines. Alternative designs using individual randomization can result in strong contamination effects or hamper proper implementation. In our design, there was no risk of contamination effects between clusters, because caregivers worked in different care organizations in different geographical regions, and did not know or meet each other during the study.

**Figure 1:**
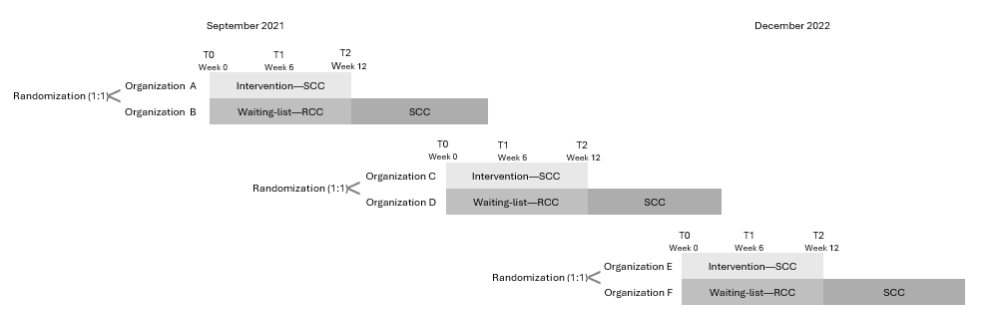
Study diagram for a staggered-entry open-label cluster randomized trial to study the effectiveness of smart continence care (SCC) compared to regular continence care (RCC).

Data were collected during 3 consecutive time points in which 6 residential care facilities for people with disabilities (clusters) were divided into 3 groups of 2 facilities (couples), refer to Figure 1. Randomization at the cluster level (1:1 ratio), executed by VJCvC and MFMG, assigned each organization within a couple to one of the 2 study groups. Allocation concealment was maintained by blinding the researcher responsible for randomization to the identity of the participating organizations at the time of allocation, which occurred at 3 separate time points (staggered entry). Before randomization, the interpretation of each possible sequence (1 to 2 vs 2 to 1) was predefined: in sequence 1 to 2, the organization labeled “1” would be allocated to the intervention group and the one labeled “2” to the waitlist group; in sequence 2 to 1, the opposite applied. The numerical labeling of the 2 organizations (“1” and “2”) was documented in advance by a separate researcher who was not involved in the randomization process, thereby keeping the randomizing researcher blinded. Randomization was conducted via an independent online tool (random.org [19]).

The main analyses of the primary and secondary outcomes were conducted by an independent statistician who was not involved in data collection and had no contact with the participating care organizations. Blinding was maintained throughout the study, group allocation was coded using a dummy variable, and the statistician was unaware of which group represented the intervention or the waiting-list group. The allocation was disclosed to the organizations (we use the term “organization” as a synonym for residential care facilities for people with disabilities) and the caregivers involved, so that the organizations allocated to the intervention group could start the preparations for implementing the SCC intervention. Given the nature of the intervention, an open-label study design was inevitable. The organizations in the waiting-list group acted as the control group, providing RCC and receiving the intervention (SCC) after their assigned data collection period.

Compared to the original trial design, as described in the protocol [13], 2 deviations had to be made due to practical circumstances. First, organization D started data collection in parallel with organizations A and B. This adjustment was made following a request from the organization’s board to initiate the research and SCC implementation earlier, to maintain momentum and motivation. Accommodating this request was considered necessary to avoid the risk of the organization withdrawing from the study a priori. Second, 2 of the organizations that initially agreed to participate were revealed to be unable to deliver eligible participants and thus could not meet the conditions for implementation. Fortunately, 2 other organizations agreed to participate. These organizations were properly randomized; however, they were not able to conduct data collection simultaneously as planned. Organization F started data collection later.

The 6 organizations that participated varied in size, number of locations housing clients, region, and client-employee ratios. The latter varied from a ratio of 2700 clients to 2700 employees to 13,500 clients to 15,800 employees. Some organizations had multiple locations across various parts of the Netherlands. The participants were located in Noord-Holland, Zuid-Holland, Gelderland, and Overijssel. For details on the recruitment, selection, and eligibility criteria of these organizations, refer to the study protocol [13].

### Ethical Considerations

#### Human Participant Ethics Review Approvals

This study was approved by the Medical Ethics Committee of Radboudumc (NL72751.091.20). If the residential care facility had a research committee, it also reviewed the research proposal and approved participation.

#### Informed Consent

Written informed consent was obtained from the legal representatives of individuals with PIMD. Caregivers consented electronically to participate in an online survey on their experiences with continence care.

#### Privacy and Confidentiality

The collected analog data were safely stored in a locked cabinet; participants’ names were removed and replaced with a pseudonymized identifier. Access to data was restricted to the researchers involved in conducting the analyses. The file containing the key linking the pseudonymized identifier with personal details was stored separately, with stricter access limited to the first author (VJCvC) and the project assistant.

#### Compensation Details

Participants did not receive any compensation for participating in the study. The residential care facilities received a €10,000 (US $10,916) reimbursement for research activities paid by the funder ZonMw.

### Comparator: RCC

The waiting-list group continued to receive RCC. RCC involved changing IMs according to a fixed schedule [5] or when caregivers identified reasons for a change, such as the detection of feces. Additional hygiene activities, such as changing bedsheets or clothes, were performed when leakage occurred. Organizations assigned to the waiting-list group kept using their regular brand of IMs and maintained their usual ways of working; only extra activities for data collection were introduced. After data collection was finalized, the organizations in the waiting-list group commenced implementation of SCC.

### Intervention: SCC

The intervention consisted of the application of SCC. Achieving this relies not only on using the new SCC technology, but also on adaptations to care and service processes. To prepare for this, the organizations in the intervention group participated in several implementation activities. Refer to our developed implementation guideline for more details [20].

For this study, the SCC system of Abena Nova (Abena A/S) was selected [13] because it was the most market-ready SCC solution available at the time of the grant application. The product consists of an IM with integrated sensors, a detachable and reusable clip, receivers, a mobile app, and an online platform. An internet connection and a mobile phone with the installed app are required. The sensor only registered urine because feces are not detectable by the technology [21]. The sensor is assumed to be highly accurate and reliable; the supplier reports 99% accuracy. However, implementation aspects, such as cleaning of the clip and network (Wi-Fi) specifications, may decrease accuracy and reliability. These issues were addressed in the training and implementation of SCC. The SCC sensor sends color-coded notifications to caregivers’ phones depending on the saturation level: change desired (orange), risk of leakage (red), or ok (green). Caregivers can then respond to these notifications by changing the IM on demand from 1 to 1.5 hours, and stop changing IMs according to fixed schedules. Thus, the intervention group was provided with different IMs, a technology that provided information about the status of the IM, and instructed on new working routines. In addition, they conducted the same data collection activities as the waiting-list group.

Without proper implementation, the effectiveness of SCC could not be investigated. Thus, we considered implementation activities as essential for the SCC group. Before providing SCC within an organization, a vast number of implementation activities took place. These included technical, organizational, and educational preparations starting in advance. Each care organization established a project team with a dedicated project leader. The care teams appointed an ambassador to function as a liaison between their care team, other care teams, the project leader, and the supplier. Ambassadors received a short explanation of SCC to help them understand the technology, and the intake was organized to select participants for the SCC. The baseline measure (T0) was conducted before the ambassadors and teams were trained in using the SCC technology. The ambassadors received in-depth training from the supplier for approximately 2 hours. Consequently, the care teams received 1 to 2 hours of training from the supplier and ambassador. e-learning was available for 30 minutes as preparation preceding the training. Before and during the implementation process, the project leader received structured implementation guidance from the research team through weekly and monthly consultation sessions [13]. While using the SCC, the care teams met regularly to discuss and evaluate the SCC system and to reach agreements on its application. The information generated by the sensor and displayed in the online portal on the voiding pattern and indication of urine volume allowed the care teams to fine-tune the SCC to an individual’s needs. During these sessions, the supplier’s incontinence specialist was present to help with the interpretation of the data and provided additional guidance on optimizing the continence care for each individual. This could include adjusting the size or absorptive capacity of the IM.

### Procedure, Participant Selection, and Eligibility Criteria

The project leader at each organization selected locations based on the potential number of eligible participants after being informed of the allocated study group. They aimed to find locations with care teams that would be able to implement SCC and execute their research efforts. After receiving an explanation of SCC, the care teams selected participants based on the eligibility criteria [13].

The inclusion criteria were a diagnosis of PIMD, age >18 years, use of regular incontinence products, inability to communicate the need to change their IMs, and signed informed consent by their legal representative. Exclusion criteria were the use of a permanent catheter or behavior that could be a risk factor for the participant when using SCC, such as a pica disorder in which the participant could swallow the clip. Inclusion was carefully considered for participants who defecated ≥3 times per 24 hours, as feces interfere with the technology detecting urine, and when there was behavior that could hinder the successful implementation of SCC, such as removal of the IMs or clips. Next, caregivers selected participants based on these criteria and the expected benefits of SCC for each individual. For example, individuals who experienced frequent bedwetting or who found a change in the IMs burdensome may benefit from changing the IMs exactly at the right moment. If required, the caregiver and supplier discussed whether providing SCC to the participants was viable. The recruitment period of the study was from August 2021 to March 2023.

After selecting participants, the caregivers informed their legal representatives, who then consented to the participation of the person with PIMD in this study. In addition, all legal representatives were invited to participate as a second proxy next to the professional caregivers by filling out quality of life questionnaires for individuals with PIMD.

### Sample Size

A sample size of 80 participants per study group and 6 clusters was deemed sufficient to detect a 20% reduction in leakage between the study groups, with a power of 80%. This calculation was based upon an intraclass correlation coefficient of 0.01, an α of .05, and a design effect of 1.05, and was subsequently confirmed through simulation efforts. Further details regarding the sample size calculation are available in the study protocol [13].

### Outcome Measures

#### Overview

The primary and secondary outcomes in both groups were measured at 3 different time points: T0, T1, and T2. For the intervention group, the care teams received training on SCC after the T0 measure, and T1 and T2 represented the use of SCC for 6 and 12 weeks, respectively. Table 1 provides an overview of the measurements over time (other outcomes described in the protocol paper will be published in due course).

**Table 1:** Overview of the questionnaires per time point.

Outcome (research instrument)		T0 (week 0)	T1 (week 6)	T2 (week 12)
**Research participants: people with profound intellectual and multiple disabilities**				
	Baseline characteristics (paper questionnaire)	✓		
	Information on continence care, such as leakage and incontinence material changes (1-wk paper continence diary)	✓	✓	✓
	Subjective well-being (MIPQ^a^)^b^	✓		✓
	Objective quality of life (QOL-PMD^c^)^b^	✓		✓
**Research participants: professional caregivers**				
	Baseline characteristics (online survey)	✓		✓
	Physical burden of giving continence care (online survey)	✓		✓

^a^MIPQ: Mood, Interest and Pleasure Questionnaire.

^b^Proxy measure, completed by caregivers on paper, for family members available as postal mail or online survey.

^c^QOL-PMD: Quality of Life for Persons with Multiple Profound Disabilities.

#### Primary Outcome: Number of Leakages

The primary outcome, the number of urinary leakages per person in 1 week, was collected using a paper continence diary. During this week, the caregivers reported instances of leakage while changing the IMs. A continence diary was specifically developed for this study and refined after pilot testing.

#### Secondary Outcomes: Number of IM Changes, Quality of Life, and Caregivers’ Physical Burden

Caregivers used a continence diary to record each instance in which the IM was changed and included information on the time of day, the reason for the IM change, and time spent on that instance of continence care. If a leakage had occurred, this included the time for additional activities, such as changing bedsheets and washing the person, and whether and for how long a second caregiver was involved. Validating these registrations with direct observations was regarded as too intruding and unethical.

Two questionnaires specifically developed for this target group were used to assess the quality of life of individuals with PIMD. With the “Mood, Interest and Pleasure Questionnaire” (MIPQ), proxies indicate the subjective well-being of a person with PIMD [22,23] based on observed mood and behavior. The questionnaire consists of 22 items with 3 subscales. Complementary to this, the “Quality of Life of Persons with Profound Multiple Disabilities” (QOL-PMD) questionnaire measures the objective quality of life [24,25], such as physical and social-emotional well-being, or the extent to which the person participates in activities. The QOL-PMD consists of 55 items (6 subscales). Both questionnaires provide a broad perspective on quality of life, assessed by a proxy for a person unable to self-report. Caregivers and legal representatives, mostly family members, served as the first and second proxies for the questionnaires, respectively.

To assess the effect of SCC on the physical burden of continence care experienced by caregivers, a web-based questionnaire was administered at T0 and T2. All caregivers involved in the project were asked to score the physical burden (1 item) of continence care (scale 0 to 10; 0=no burden and 10=very high burden).

### Analysis

#### Main Analysis

An intention-to-treat analysis was performed in which we included all participants who were rightfully included after randomization, formally known as a modified intention-to-treat analysis [26]. The primary outcome, the number of leakages per week for each time point, and the secondary outcomes, the number of IM changes per week, quality of life, and caregivers’ physical burden, were originally planned to be analyzed using a generalized linear mixed model (GLMM) with a Poisson link function (R version 4.0+, package lme4 [27]; R Foundation for Statistical Computing) containing 3 levels: repeated measurements (level 1), nested within participants (level 2), who were themselves nested within disability care organizations (level 3). However, we decided to include a random intercept for location instead of organization, as the residential care teams worked on a per-location basis.

The effect of the intervention at each time point was assessed through interaction terms with dummy variables denoting each time point, as described by Twisk et al [28] (excluding the fixed effect of the intervention), and repeated measurements within participants, as well as nesting at the location level, were addressed through random intercepts. The advantage of using the GLMM is that it includes incomplete cases in the analysis and uses restricted maximum likelihood estimation to calculate parameter estimates. Hence, no additional imputation is required for completely missing participant data points [29].

As opposed to what was described in the original protocol [13], a Poisson link function was not used. Missing days within measurement weeks (ie, time points in the model) were addressed by extrapolating the number of leakages and IM changes to 7 days (for example, if 2 leakages had occurred on 3 days, while 4 days were not registered, the number of leakages was set to 2/3×7=4.67 for that particular participant at that time point). Extrapolating these values resulted in noninteger values for the outcomes per week; hence, Poisson regression was no longer appropriate. The weekly duration of continence care per time point was determined by first calculating each individual’s average duration of one continence care moment at T0, T1 and T2 and then multiplying it by the participant’s extrapolated number of weekly IMs for that measurement.

The statistician (WdH) was blinded to the randomization outcome for each organization until the main analyses were conducted. The same GLMM model structures were used for the primary and secondary outcomes.

Furthermore, per-protocol and completer analyses were performed to determine the number of leakages and IM changes. Per-protocol adherence was defined as adherence to the study protocol. In the waiting-list group, adherence was defined as wearing regular IMs at T2. For the intervention group, this meant wearing smart continence materials at T2. Periods of temporary discontinuation of smart continence materials, such as those caused by stock shortages, were allowed as long as there had not been a decision to completely stop using SCC for this participant. A completer was defined as a participant having their continence care registered for 7 days at each time point.

#### Exploratory Analyses

An exploratory analysis was added to the analysis described in the protocol. The goal was to examine data on the number of leakages and the number of IM changes per organization and location to understand why intention-to-treat analyses revealed an unexpected result (refer to the Results section). Combined with our knowledge of the implementation struggles experienced by a specific organization, we decided to repeat the GLMM analyses on the number of leakages and IM changes after excluding this particular organization.

## Results

### Data Collection, Data Entry, and Response Rates

A total of 34 organizations were contacted, and 6 (18%) of them were recruited into this study. A total of 29 locations (residences) participated across the 6 organizations, ranging from 2 to 7 locations per organization, with 2 to 12 participants per location, and an average of 5.4 (SD 2.44) participants per location.

**Figure 2:**
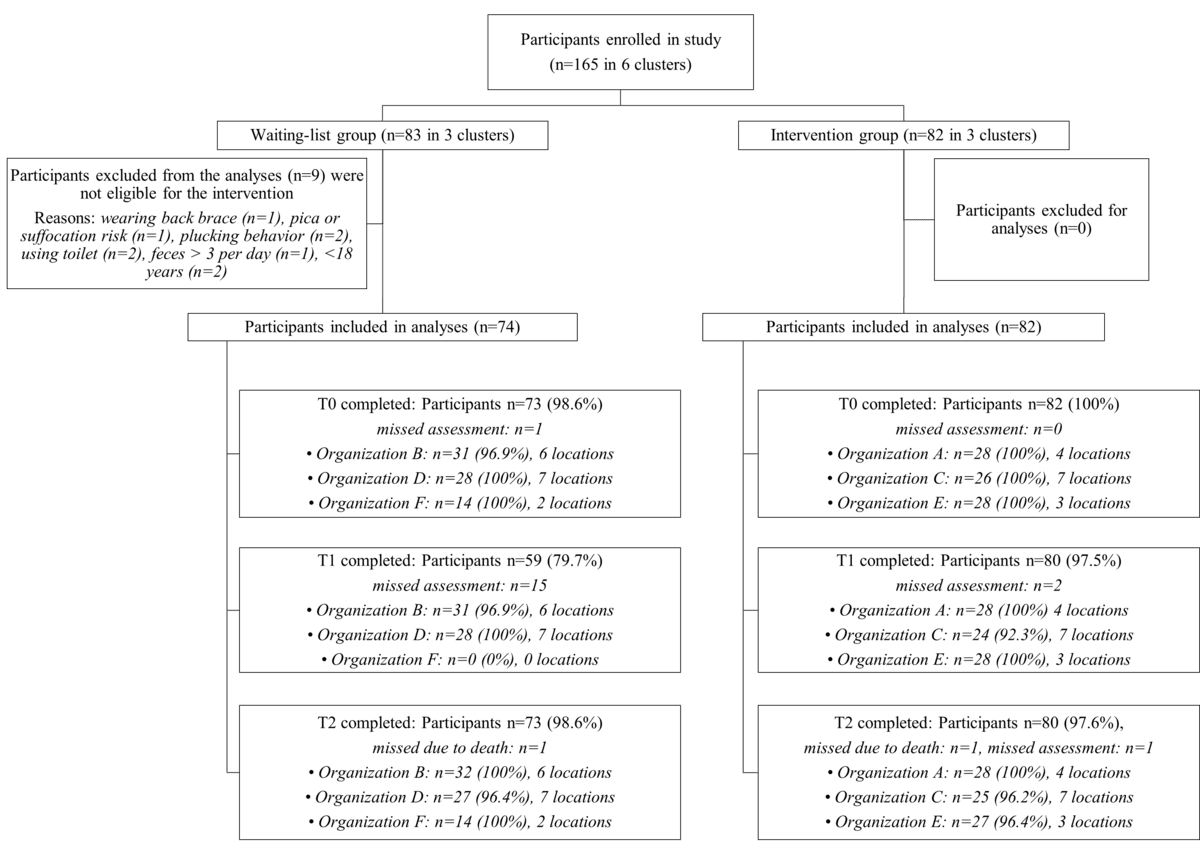
Flowchart demonstrating the number of participants randomized at the cluster level, including the response rates (%) for the outcome measures: the number of leakages, the number of incontinence material changes per week, and the time spent on continence care.

A total of 165 participants were enrolled in the study, and data from 156 (94.5%) participants were included in the primary analyses. Within the waiting-list group, after data collection at T2, 11% (9/82) participants did not meet the eligibility criteria for SCC. The data collected from these participants were removed from the dataset and excluded from the study (Figure 2).

Almost all 1-week paper continence diaries (447/468, 95.5%) were returned with at least some records of registered continence care. In 1.3% (117/9113) of the registered instances of continence care, there was no information on whether a leakage had occurred, as this question was left empty. These instances were handled as “no leakage.” One organization (organization F) did not participate in the T1 measure. This was done to prevent them from dropping out because of their request to lower the workload of their research activities. According to the intention-to-treat principle, all returned diaries were included in the analyses, including the records of continence care extrapolated to 1 week.

The caregiver and close relatives completed quality-of-life questionnaires (MIPQ and QOL-PMD) at T0 and T2. The caregivers’ overall response rate to these questionnaires was 84.1% (525/624 questionnaires). Due to incomplete responses, 4.8% (13/269) of the returned MIPQ did not provide a total score. For the QoL-PMD questionnaire, the rate was 37.1% (96/259). Only 5.8% (18/312) of the expected quality-of-life questionnaires were completed and returned by relatives at T0 and T2. Therefore, the responses from relatives were excluded from the analysis. Thus, the participants’ quality-of-life scores were based solely on the caregivers’ answers.

Analogously collected data, that is, the pen and paper diaries and questionnaires, were entered into Microsoft Excel (version 16.0) by the researchers and research assistants under the supervision of the researchers. VJCvC and an independent researcher conducted a quality check and found <1% incorrect inputs, which was deemed acceptable [30].

The overall response rate for caregivers’ experiences of the physical burden of continence care completed at T0 and T2 with an online survey was 48.3% (182/377). At T0, the response rates were 51% (126/247) in the intervention group and 43.1% (56/130) in the waiting-list group. At T2, these rates dropped to 32.2% (86/267) in the intervention group and 27.3% (71/260) in the waiting-list group.

### Baseline Characteristics

Table 2 shows the baseline characteristics of participants with PIMD. Their average age was 48.7 (SD 14.9) years. Approximately half of the participants (86/156, 55.1%) were men. The vast majority (118/136, 86.8%) had a developmental age of <4 years. The table also shows comorbidities and mobility classes among the participants. Both groups were equally distributed in terms of sex, age, and developmental age; however, they appeared to differ, at face value, in the degree of mobility and presence of comorbidities.

Table 3 shows caregivers’ characteristics, as reported in an online survey measuring the physical burden during continence care.

**Table 2:** Baseline characteristics of the participants with profound intellectual and multiple disabilities.

Variable			Waiting-list group		Intervention group
Age (years), mean (SD; range)			45.4 (14.3; 19-75)^a^		51.7 (14.9; 20-80)^b^
**Sex, n/n (%)**					
	Female	35/74 (47)		35/82 (43)	
	Male	39/74 (53)		47/82 (57)	
**Developmental age range, n/n (%)**					
	0-6 months	17/70 (24)		26/66 (39)	
	6-12 months	30/70 (43)		19/66 (29)	
	12-18 months	4/70 (6)		11/66 (17)	
	18-24 months	4/70 (6)		1/66 (2)	
	2-4 years	5/70 (7)		1/66 (2)	
	4-8 years	2/70 (3)		1/66 (2)	
	Unable to assess	8/70 (11)		7/66 (11)	
**Mobility class^c^, n/n (%)**					
	A	1/63 (2)		10/76 (13)	
	B	6/63 (10)		18/76 (24)	
	C	5/63 (8)		7/76 (9)	
	D	33/63 (52)		33/76 (43)	
	E	18/63 (29)		8/76 (11)	
**Comorbidities, n/n (%)**					
	Epilepsy	46/74 (62)		42/81 (52)	
	Swallowing problems	40/74 (54)		32/81 (40)	
	Urine tract infections	16/74 (22)		11/81 (14)	
	Incontinence-associated dermatitis	18/74 (24)		9/81 (11)	
	Skin problems	35/74 (47)		35/81 (43)	
**Dutch care profile^d^ n/n (%)**					
	5VG	11/71 (16)		21/80 (26)	
	7LG	12/71 (17)		11/80 (14)	
	8VG	45/71 (63)		41/80 (51)	
	Other	3/71 (4)		7/80 (9)	

^a^n=74.

^b^n=82.

^c^Mobility classes [31] were defined as follows: class A—the client can perform the task independently, with or without aids or special adaptations; class B—the client cannot perform the task independently, but the required assistance does not pose a risk of physical strain for the caregiver; class C—the client requires assistance, and the caregiver must physically support the transfer to ensure safety; class D—the client cannot perform the task independently and requires a mechanical aid, such as a patient hoist; and class E—the client is entirely dependent on the caregiver for transfers and cannot play an active role.

^d^The Center for Indication of Care Needs (CIZ, Centrum Indicatiestelling Zorg) assigned these profiles to individual clients to ensure appropriate care. These 3 care profiles were indicated to be the most likely for people with profound intellectual and multiple disabilities [32]. 5VG (intellectual disability): this profile was designed for individuals who required intensive guidance and care. It is suitable for people with intellectual disabilities who need substantial support in their daily lives. 7LG (physical disability): this profile involves living in an open or closed setting with very intensive guidance, care, and behavior regulation. It was designed for individuals with mild intellectual disabilities and complex care needs. 8VG (intellectual disability): this profile was for people who require full care, including nursing and support. It was designed for individuals with intellectual disabilities who need intensive nursing and care.

**Table 3:** Characteristics of the caregivers by study group per time point.

Characteristics		Waiting-list group			Intervention group	
		Time point: T0 (n=56)^a^	Time point: T2 (n=71)^b^	Time point: T0 (n=126)^c^		Time point: T2 (n=86)^d^
**Sex, n (%)**						
	Female	51 (91)	64 (90)	114 (90)		74 (86)
	Male	4 (7)	6 (8)	12 (10)		10 (12)
	Wish to not say	1 (2)	1 (2)	0 (0)		2 (2)
**Age group (years), n (%)**						
	<18	1 (2)	1 (1)	1 (1)		0 (0)
	18-25	8 (14)	6 (8)	11 (9)		10 (12)
	26-35	10 (18)	14 (20)	29 (23)		18 (21)
	36-45	12 (21)	17 (24)	29 (23)		17 (20)
	46-55	12 (21)	14 (20)	32 (25)		24 (28)
	≤56	12 (21)	18 (25)	24 (19)		14 (16)
	Do not wish to say or missing	1 (2)	1 (1)	0 (0)		3 (3)
**Working at^e^ n(%)**						
	Residence	37 (66)	45 (63)	75 (60)		54 (63)
	Day activity center	16 (29)	15 (21)	29 (23)		16 (19)
	Night care	8 (14)	17 (24)	26 (21)		17 (20)
	Other	1 (2)	2 (3)	0 (0)		0 (0)

^a^Invited (n=130); responded (n=56, 43%).

^b^Invited (n=260); responded (n=71, 27%).

^c^Invited (n=247); responded (n=126, 51%).

^d^Invited (n=267); responded (n=86, 32%).

^e^A caregiver can work in multiple teams.

### Primary Outcome: Number of Leakages

**Figure 3:**
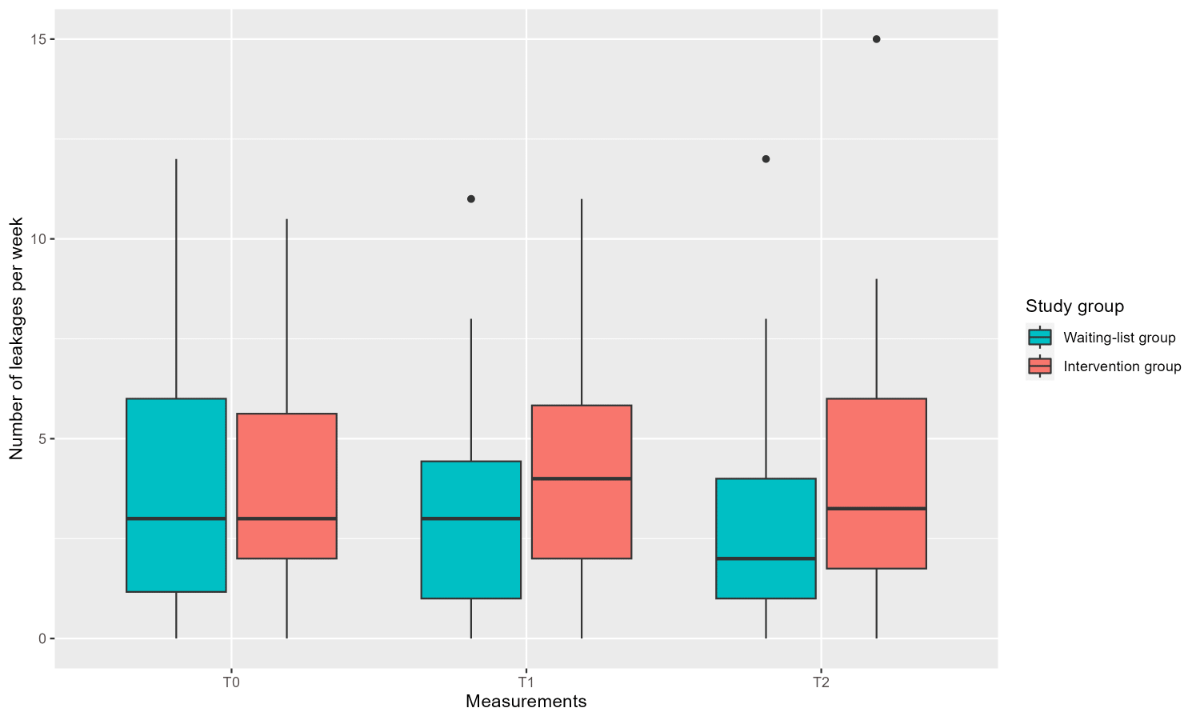
Boxplot showing the number of leakages per week at the 3 time points for each study group.

The waiting-list group showed a greater decrease in the number of leakages per week between T0 and T2 (29%; T0: mean 3.81, SD 3.03; T2: mean 2.71, SD 2.34) than the intervention group, which showed no change (Figure 3; Table 4). This is reflected by the β coefficient of 1.013 (95% CI 0.217-1.808; *P*=.01) at T2 with a small effect size (Cohen *d*=0.29, 95% CI 0.06-0.51) [33] (Table 5).

**Table 4:** Primary and secondary outcome measures during 3 time points by study group—intention-to-treat analysis.

Outcome			Waiting-list group, mean (SD)						Intervention group, mean (SD)				
			T0		T1		T2		T0		T1		T2
**Primary outcome**													
	Leakages per week	3.81 (3.03)^a^		3.24 (2.62)^b^		2.71 (2.34)^a^		3.78 (2.67)^c^		3.95 (2.83)^d^		3.83 (2.81)^d^	
**Secondary outcomes: continence care**													
	Incontinence material changes per week	20.94 (6.29)^a^		19.46 (6.73)^b^		18.14 (5.59)^a^		20.29 (4.28)^c^		16.36 (4.48)^d^		15.71 (4.88)^d^	
	Duration (min) per incontinence material change	15.60 (5.63)^a^		15.63 (6.65)^b^		14.56 (6.66)^a^		12.12 (7.70)^c^		13.10 (7.82)^d^		12.19 (7.72)^d^	
	Duration (min) for continence care per week	318.36 (127.61)^a^		292.60 (125.30)^b^		257.60 (130.22)^a^		238.15 (146.31)^c^		208.74 (138.81)^d^		185.52 (117.55)^d^	
**Secondary outcomes: quality of life**													
	Subjective well-being (MIPQ^e^)	47.64 (12.18)^f^		N/A^g^		48.39 (12.25)^h^		50.16, (11.69)^i^		N/A		47.98 (11.22)^j^	
	Objective well-being (QOL-PMD^k^)	54.84 (13.91)^l^		N/A		53.25 (10.55)^m^		56.08 (14.33)^n^		N/A		54.33 (12.68)^o^	

^a^n=73.

^b^n=59.

^c^n=82.

^d^n=80.

^e^MIPQ: Mood, Interest and Pleasure Questionnaire; scores ranging from 0 to 88, with higher scores indicating better subjective well-being.

^f^n=57.

^g^N/A: not applicable.

^h^n=54.

^i^n=78.

^j^n=67.

^k^QOL-PMD: Quality of Life of Persons with Profound Multiple Disabilities, score ranging from 0 to 100, with higher scores indicating better objective quality of life.

^l^n=38.

^m^n=21.

^n^n=49.

^o^n=55.

**Table 5:** Outcome linear mixed model intention-to-treat analyses of primary and secondary outcomes, comparing the intervention group to the waiting-list group.

Outcomes			β coefficient (SE; 95% CI)	*P* value	Cohen *d* (95% CI)
**Primary outcome**					
	**Leakages per week**				
		Mean difference: T0 – T1	0.83 (0.43; −0.01 to 1.66)	.05	0.22 (−0.001 to 0.45)
		Mean difference: T0 – T2	1.01 (0.41; 0.22 to 1.81)	.01	0.29 (0.06 to 0.51)
**Secondary outcomes**					
	**Incontinence material changes per week**				
		Mean difference: T0 – T1	−2.02 (0.69; −3.37 to −0.66)	.004	−0.33 (0.55 to 0.11)
		Mean difference: T0 – T2	−2.00 (0.66; −3.30 to −0.71)	.003	−0.34 (0.57 to −0.12)
	**Duration for continence care per week (min)**				
		Mean difference: T0 – T1	−2.27 (16.64; −34.88 to 30.34)	.89	−0.02 (−0.24 to 0.21)
		Mean difference: T0 – T2	3.29 (15.87; −27.80 to 34.39)	.84	0.02 (−0.20 to 0.25)
	**Subjective well-being (MIPQ)^a^**				
		Mean difference: T0 – T2	−0.80 (1.83; −4.39 to 2.78)	.66	−0.07 (−0.40 to 0.25)
	**Objective well-being (QOL-PMD)^b^**				
		Mean difference T0 – T2	4.73 (2.68; −0.52 to 9.98)	.08	0.41 (−0.05 to 0.87)

^a^MIPQ: Mood, Interest and Pleasure Questionnaire.

^b^QOL-PMD: Quality of Life of Persons with Profound Multiple Disabilities questionnaire.

### Secondary Outcomes: Number and Duration of IM Changes, Quality of Life, and Caregivers’ Physical Burden

**Figure 4:**
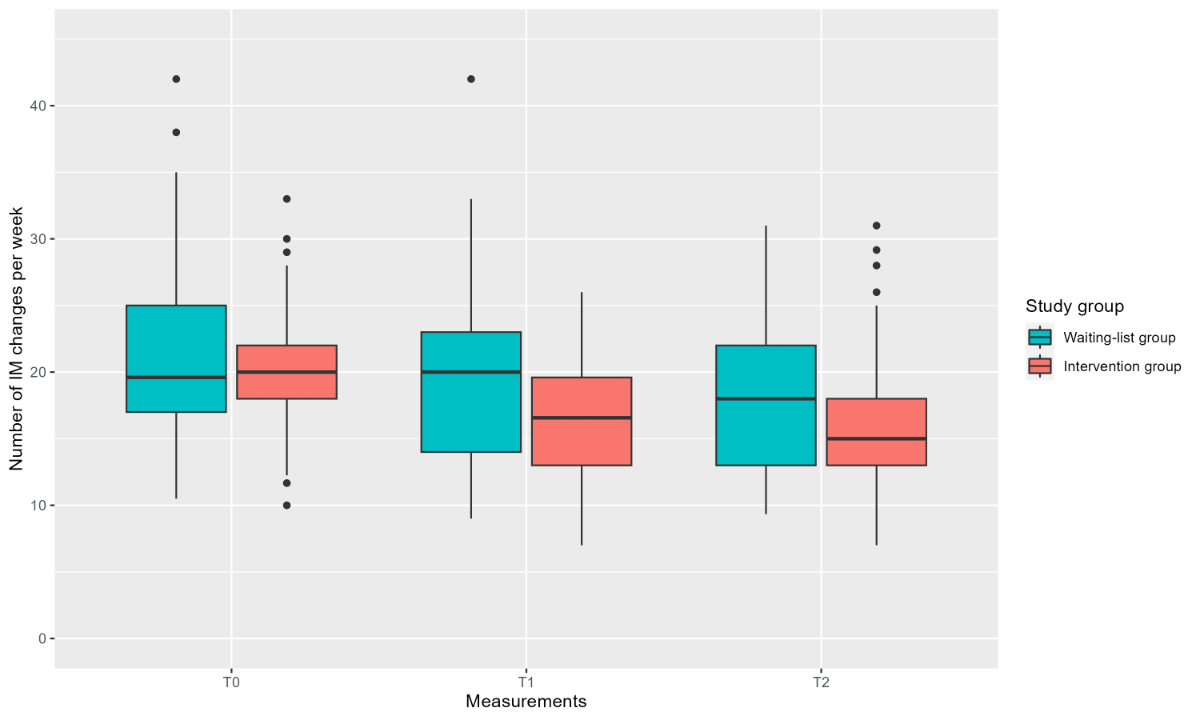
Boxplot showing the number of incontinence material (IM) changes per week at the 3 time points for each study group.

Analyses of the number of weekly IM changes revealed a significant effect (*P=*.003) in favor of the intervention group, with a small effect size (Cohen *d=*−0.34, 95% CI −.57 to −.12). This means that the number of IM changes decreased by 2 per week more in the intervention group using SCC compared to RCC in the waiting-list group (Table 5; β coefficient=−2.005, 95% CI −3.303 to −0.707 at T2). The average number of IM changes per week at T0 was 20.29 (SD 4.28) and 20.94 (SD 6.29) in the intervention and waiting-list groups, respectively. For the intervention group, the number of IM changes per week decreased to 16.36 (SD 4.48) at T1 and 15.71 (SD 4.88) at T2, whereas the waiting-list group showed a smaller decrease, 19.46 (SD 6.73) at T1 and 18.14 (SD 5.59) at T2 (Figure 4; Table 4).

**Figure 5:**
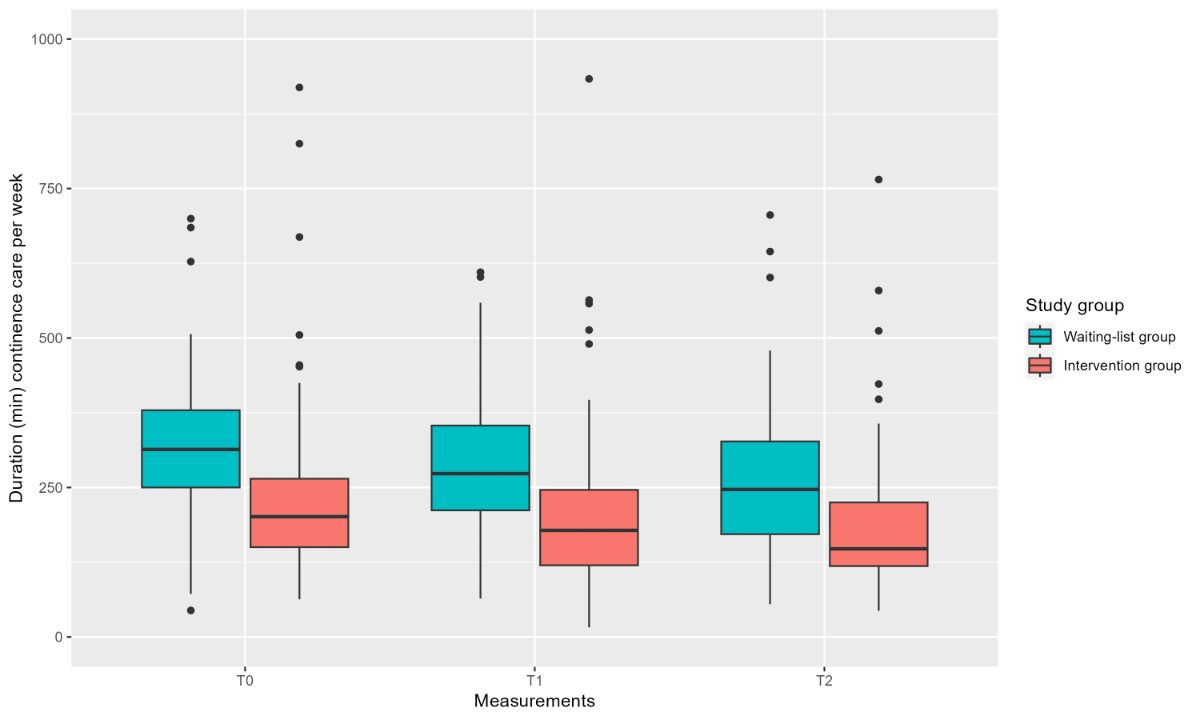
Boxplot showing duration of continence care and related activities (in min/wk) at the 3 time points for each study group.

The average time spent providing continence care per week at T0 was 238.15 minutes (ie, approximately 4 h, SD 146.31 min, ie, approximately 2.4 h) for the intervention group, and 318.36 minutes (ie, approximately 5.3 h, SD 127.61 min, ie, approximately 2.1 h) for the waiting-list group. At T2, the average time spent providing continence care was 185.52 minutes (ie, approximately 3.1 h, SD 117.55, ie, approximately 2 h) for the intervention group and 257.60 minutes (ie, approximately 4.3 h, SD 130.22, ie, approximately 2.2 h) for the waiting-list group. Approximately 10% of IM changes required help from a second caregiver. Figure 5 shows the total time spent on continence care per week based on the average time per IM change and the extrapolated number of IM changes per week. Although there was no significant difference between the groups over time (*P*=.84; Table 5), both groups did decrease their time spent on continence care by an average of 1 hour.

Scores in the quality-of-life measures (QOL-PMD and MIPQ) showed no significant differences between the groups over time (Table 5; *P=*.08 and *P*=.66, respectively).

The average score of caregivers’ physical burden regarding continence at T0 was 5.51 (SD 2.43) for the waiting-list group and 6.23 (SD 2.04) for the intervention group. A higher score indicated a higher burden. After 12 weeks, the physical burden slightly improved for both groups: 5.17 (SD 2.31) and 5.38 (SD 2.38) for the waiting-list and intervention groups, respectively. Overall, no significant effect (*P*=.09) was observed (β coefficient= −0.65, 95% CI −1.39 to 0.09; Cohen *d=*−0.34, 95% CI −0.73 to 0.04).

### Per-Protocol and Completer Analyses

In total, 23 (28%) of the 82 participants stopped receiving SCC within 12 weeks. At one location, legal representatives requested that the SCC be stopped because of increased nocturnal leakage. In addition, caregivers were dissatisfied with the absorptive capacity and fit of the IM and experienced difficulties in adjusting the timing of IM changes. This led to the decision to discontinue the use of SCC after 4 weeks for all participants (8/23, 35%) at this location. For 39% (9/23) of the participants, specific reasons for discontinuing SCC were mentioned. One participant died (4%), 3 (13%) participants stopped because of skin problems, 4 (17%) participants did not accept the clip or the changing schedule that came with SCC, and 1 (4%) participant voided large amounts of urine at once, which the IM could not contain. For this participant, receiving SCC did not provide additional benefits, except for this insight. For 17% (4/23) of the participants, multiple reasons for discontinuation were mentioned, including skin problems (2/23, 9%), rejection or removal of clips or IMs by the participants (3/23, 13%), additional stress due to the duration of IM change (1/23, 4%), increased number of leakages or loose fit (3/23, 13%), and caregivers not adjusting their work routines and changing the IMs before notification (1/23, 4%). For 9% (2/23) of the participants who discontinued, the reasons for discontinuation were not documented. In the waiting-list group, one participant passed away.

The per-protocol analysis (n=132; waiting-list group n=73, 55.3%; intervention group n=59, 44.7%) yielded similar results to the intention-to-treat analyses, with a significant β coefficient of 1.00 for leakages (*P=*.02) and similar effect size (Cohen *d*=0.28, 95% CI 0.03-0.53), as well as a significant β coefficient of −2.08 for IM changes (*P=*.003) with an almost similar effect size (Cohen *d*=0.37, 95% CI 0.13-0.61) at T2.

A total of 62 participants were included in the completer analyses: 34 (55%) participants in the intervention group and 28 (45%) participants in the waiting-list group from 18 locations in 5 organizations. In this analysis, the number of leakages no longer revealed a significant effect. Although in the intention-to-treat analyses the number of leakages showed a larger decrease over time in the waiting-list group as compared to the intervention group, the completer analysis showed no significant difference between the groups (β coefficient=1.35; *P=*.06). However, regarding the number of IM changes, the completer analysis revealed a larger decrease in the number of IM changes (β coefficient from −2.00 to −2.62) in the intervention group as compared to the waiting-list group, with a greater effect size (Cohen *d* −0.34 to −0.44 and corresponding 95% CI −0.78 to −0.10).

### Exploratory Subgroup Analysis

**Figure 6:**
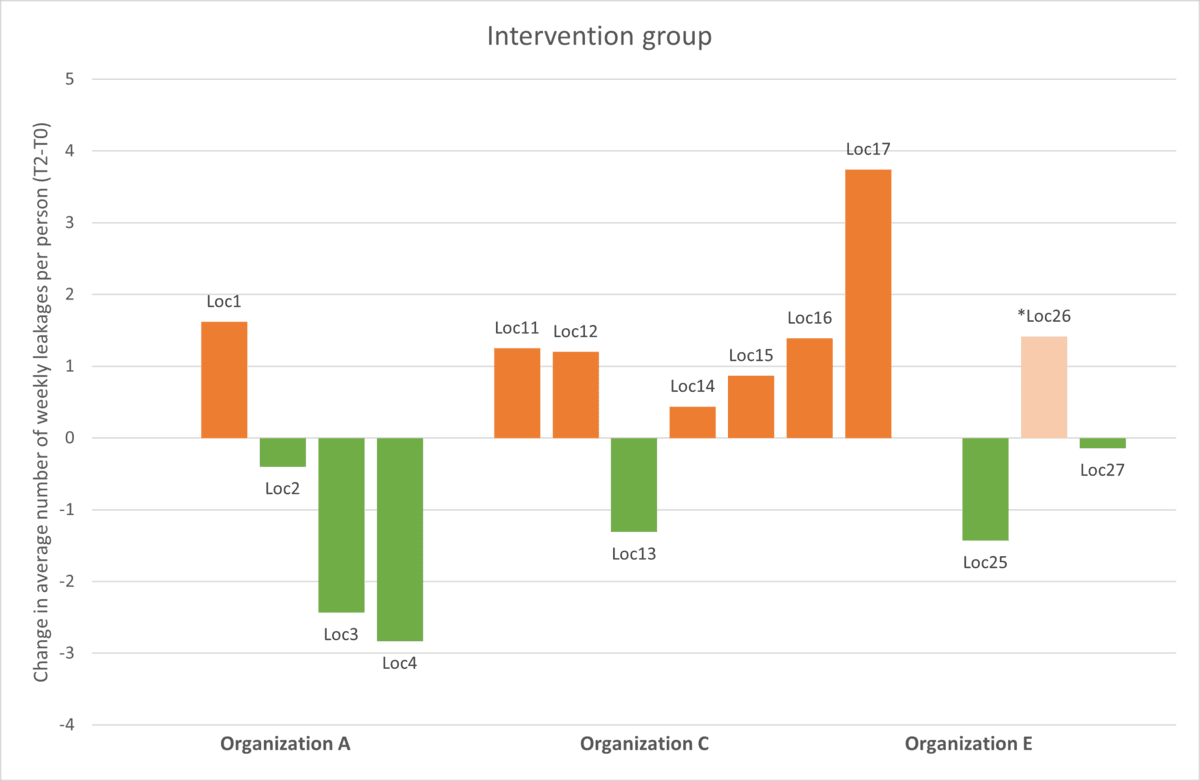
The change in the average weekly leakages per person, grouped per location, for the intervention group. Orange indicates an increase and green indicates a decrease. *One location at organization E, which stopped smart continence care after 4 weeks, shows data from regular continence care at T2.

**Figure 7:**
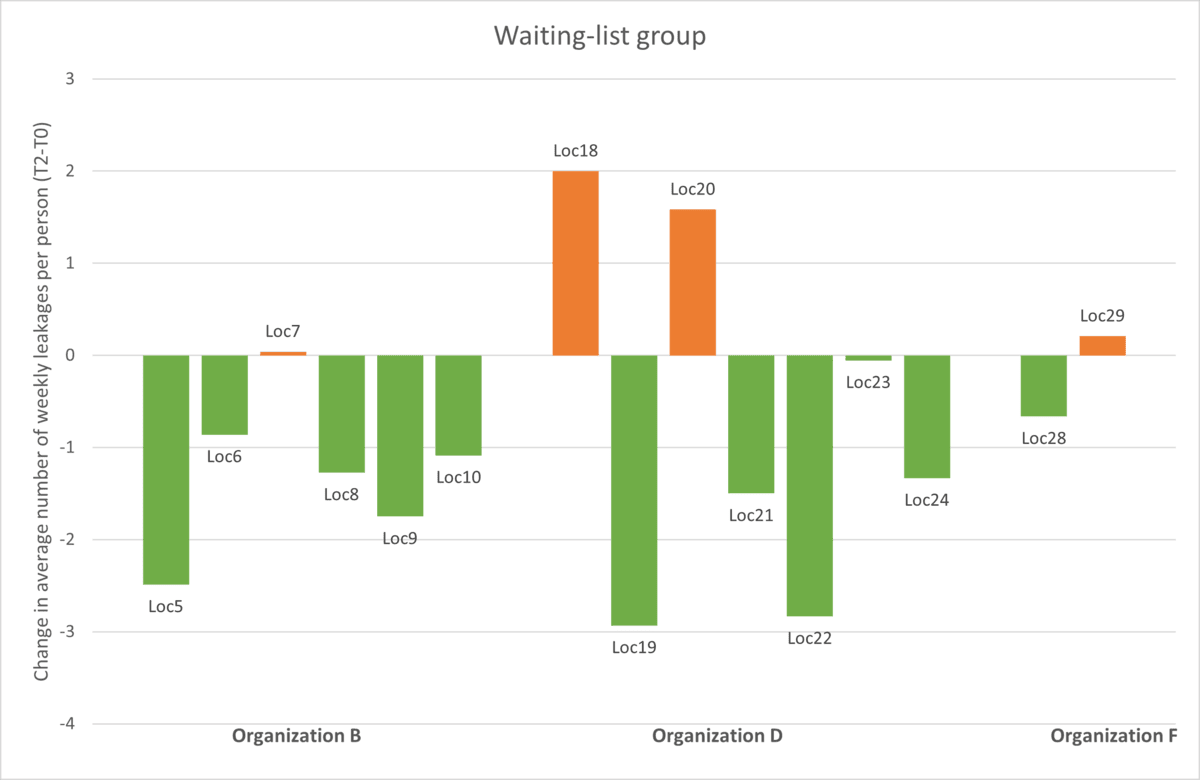
The change in the average weekly leakages per person, grouped per location, for the waiting-list group. Orange indicates an increase and green indicates a decrease.

Observations during the study provided input for the exploratory subgroup analysis. Organizations must overcome several barriers when implementing SCC. Therefore, we chose to explore the changes in leakage and IM per location (Figures 6 and 7) for all participants. This showed that the locations in organization C experienced an increase in the number of leakages per week over time. Combined with our knowledge that this organization’s project leader reported significant difficulties with staff turnover, resulting in less successful implementation of SCC, it was decided to repeat the GLMM analysis, excluding organization C.

The exploratory per-protocol GLMM analysis excluded organization C (n=113 participants included). The analysis showed that the average number of leakages per week in this subintervention group reduced from 4.56 (SD 2.90) at T0 to 3.65 (SD 2.35) at T2, and the waiting-list group showed a reduction from 3.78 (SD 3.04) at T0 to 2.71 (SD 2.34) at T2. The β coefficient (0.30) was no longer significant (*P=*.54), and there was no significant effect size (Cohen *d*=0.09, 95% CI −0.19 to 0.36). With this analysis, the unexpected result in the primary outcome disappeared. The effect size for the number of IM changes increased from Cohen *d* −0.34 to −0.45 (95% CI −0.71 to −0.18) with a β coefficient of −2.50 that remained significant (*P=*.001), supporting our previous observation on this measure.

## Discussion

### Principal Findings

This study is the first to evaluate the effectiveness of SCC in residential care facilities for individuals with PIMD using a cluster randomized trial with a large sample size (n=156). Research on the effectiveness of care technologies that could enhance the lives of individuals with PIMD is crucial, as they need support in every aspect of their lives and spend most of their lives in residential care facilities.

The intention-to-treat and per-protocol analyses revealed a significant and unexpected effect on the primary outcome: the number of leakages. There was a larger reduction in the number of weekly leakages in the waiting-list group as compared to the intervention group, in which the number of leakages remained the same at all time points. However, in the analyses of the study completers, this difference between the 2 groups was no longer significant. Moreover, there was no longer a difference between the mean differences over time in the primary outcome for both groups in the exploratory subgroup analysis.

For the secondary outcome, the number of weekly IM changes, we found the results as hypothesized, showing a reduction over time in the intervention group that was significantly larger than the observed decrease in the waiting-list group. The effect size was larger in the completer and exploratory subgroup analyses. No significant reduction in time spent on continence care was observed, and there was no effect on the quality of life, nor did we find an improvement in caregivers’ physical burden.

How should the findings be interpreted? Our study did not find that SCC was more effective than RCC in reducing the number of leakages. In the exploratory subgroup analysis, the mean number of leakages decreased in both groups. This might be a consequence of the fact that in both groups, attention was paid to the continence care process. The care process, urine patterns, number of IM changes, and leakages of all participating individuals with PIMD were monitored in diaries as part of the trial. It is likely that this attention would have improved continence care itself, whether technology is applied or not. This may suggest the Hawthorne effect [34], which refers to the phenomenon in which individuals modify their behavior in response to being studied, which can influence study outcomes.

Our study revealed that SCC was effective in reducing the number of IM changes, and this effect appeared stronger in the completer and exploratory subgroup analyses, indicating the robustness of this finding. This is an important finding as individuals with PIMD then experience fewer disturbances in their daily activities or at night, which reduces workload for caregivers. Contrary to our expectations, however, our study did not reveal that SCC was more effective than RCC in reducing the time spent on continence care. In fact, the time spent showed a reduction in both groups, but this did not significantly differ between the groups.

The fact that the effect size for the reduction in the number of IM changes because of SCC grew stronger in the completers analyses despite the smaller number of included participants might indicate the team’s capability to complete the research efforts, as caregivers are responsible for a participant being a completer, which may be related to the more positive outcomes associated with SCC. It seems that teams that were able to fill out the diaries each day during all 3 measurement weeks (ie, completers) were better able to improve their continence care while using SCC technology. This suggests that a team’s capabilities play a role in the extent to which they benefit from the potential of SCC technology.

Our observations also support the importance of good implementation. While all organizations received implementation guidance, some faced more barriers than others. During the consultation sessions of our research team with organization C, we discussed their struggles with their high staff turnover and their dependence on flexible workers. Furthermore, immediately before they started implementing SCC, one of the 2 project leaders left the organization. In addition, the project leader was also in charge of coordinating the COVID-19 pandemic measures within the organization, which took priority over the implementation of SCC. It can be expected that these barriers are part of the explanation for the increased leakage among the participants of this organization. Working with new technology and changing work routines complicate continence care. The combination of the aforementioned struggles led to the less successful implementation of SCC in this organization.

Previous studies on technology monitoring continence care in residential care for older adults focused on timely and prompted toilet use or right-on-time changes in saturated IMs, have shown mixed nongeneralizable results compared with our study. Nikoletti et al [35] compared personalized toilet routines based on electronic and manual assessments for older adult patients in an acute care setting in Australia. A significant within-group leakage reduction was observed in the intervention group but not in the control group. However, between-group differences were not statistically significant. The generalizability of their study is limited owing to its small sample size (n=41) and minimal completed data (n=6). A Canadian group [7] with a larger sample (n=101) of older nursing home residents conducted a cluster randomized trial (cluster being at the unit level) focusing on personalized continence care plans constructed with and without electronic continence monitoring. No significant between-group differences in leakage reduction were found, similar to our results. However, a significant within-group leakage reduction was observed in the intervention group. Exploring their numbers might suggest an irreducible number of leakages in a normal care setting. It is not unlikely that their waiting-list group already had this “minimum level of leakages” (around 2.45 per week), whereas their intervention group had some room to improve (from 3.78 to 2.10 leakages per week). Agholme et al [6] reported a lower minimum level of leakage per week (1.4) for Polish older people, which was reached when using a product similar to that used in our study. However, this number should be interpreted carefully, as the sample size was small (n=35) and the setting was different (home environment). They found a significant within-group decrease in the number of leakages, but no significant decrease in the number of IM changes. As indicated, all these studies were conducted in older people. Care for people with disabilities is often organized differently, and differences may exist between nations. Furthermore, differences exist between older people and people with PIMD in terms of their medical and physical condition, their health status over time, and their level of activity during the day. Our study answers questions regarding the effectiveness of SCC for people with PIMD living in Dutch residential care facilities for people with disabilities.

Each improvement in the care of individuals with PIMD is crucial, given their heavy dependence on caregivers and limited ability to improve their lives. However, contrary to our expectations, SCC was not effective in improving the quality of life of participants with PIMD. It is possible that changes in quality of life could not be detected using the questionnaires used in our study, or that our sample size was too small to detect differences in quality of life over time. Our observations during the study indicate that SCC technology can have positive effects on individuals with PIMD when their care teams can incorporate these insights and adjust their work routines accordingly. However, 28% (23/82) of participants in the intervention group stopped SCC before the end of the data collection due to negative experiences, such as increased leakages, skin issues, or clip rejection. The caregivers noted that the IM sometimes did not fit well due to unique postures resulting from physical disabilities. Some participants rejected the clip because of cognitive disabilities, occasionally hiding or removing it. These findings underscore the need for careful consideration of which individuals might benefit from SCC. Organizations should follow eligibility criteria, but may consider a try if no harm is expected (eg, absence of pica behavior). The benefits and potential risks of SCC should be closely monitored, while the data collected on the participants’ voiding patterns can provide valuable insights to further personalize and improve their continence care. These mixed results warrant further investigation.

### Strengths and Limitations

Our study had several strengths. To the best of our knowledge, this was the first cluster randomized trial to examine the effectiveness of SCC in the residential care of individuals with PIMD. Conducting such research with participants with PIMD was unique because it is demanding and time-consuming to collect data in a multicenter trial in this setting. A key strength of the study was that, despite the COVID-19 pandemic that forced us to postpone part of the data collection and the fact that 2 of the initial care facilities decided to withdraw their participation in the study, we managed to reach the target number of organizations (n=6) and number of participants (n=156) needed according to the power calculation [13]. Our study was well-powered and conducted according to a previously published research protocol and CONSORT guidelines. The response rates, especially for the main outcome measures, were high, and the dropout rate was low. The data analyses were performed using GLMM and a blinded assessor, considering interaction effects and comparing the mean difference over time in each study group. We did not apply formal corrections (eg, Bonferroni or Holm-Sidak) for multiple testing, as the primary and secondary outcomes are conceptually related and thus not independent. Corrections assuming independence would be overly conservative. Nonetheless, even under a Bonferroni correction (adjusted significance level 0.0125), the pattern of significant results at T2 remains unchanged, supporting the robustness of our conclusions.

Furthermore, utmost attention has been paid to the implementation of the intervention. Studying the effectiveness of integrating technological solutions into residential care is only possible if the implementation of the technology, including changing the way of working in daily routines, is a key part of the project [36,37]. Our team developed an implementation guideline [20] for residential care facilities for people with disabilities and guided project leaders and care teams at every implementation step. For example, we provided weekly consultations to the project leaders of organizations and care teams received support from the supplier’s continence care expert. The close involvement of our research team ensured a rich understanding of the collected data, including the stories behind them. While considerable efforts were made to ensure intervention fidelity, it is important to acknowledge that contextual factors may still have influenced adherence. A strength of our approach is also that these influences are reflected in the per-protocol, completer, and exploratory analyses and are illustrative of real-world practice.

However, several limitations of this study should be considered when interpreting our results. The original research plan [13] called for simultaneous data collection within each pair, which was only conducted successfully in 1 of the 3 pairs. However, we suspect that this did not affect our analyses, as we compared the aggregated data for the intervention and waiting-list groups, and the data were still collected equally in the first and second halves of the year for both groups.

Blinding was not feasible due to the nature of the intervention. Although caregivers were aware of group allocation, the risk of detection bias was limited due to the objective nature of the outcomes, standardized instructions across groups, and the absence of incentives to influence reporting. Moreover, participant selection reflected usual care practices, and all participants eventually received the intervention.

Another limitation was the lack of access to the data gathered by the smart continence sensor, which could have acted as a quality check on the manual record in paper diaries. As the number of leakages and IM changes is extrapolated, this may have led to either an under- or overrepresentation of these numbers. This applied to both groups. Therefore, the β coefficients and effect sizes found in the analyses are representative.

Although our study showed a low dropout rate among the participants with PIMD, the response rates for the caregiver questionnaire assessing the physical burden they experienced related to the continence care they provided were relatively low. Therefore, the results should be interpreted with caution.

Two questionnaires were used to measure the quality of life of individuals with PIMD. The MIPQ [22] was used for subjective well-being, and the QOL-PMD [24] was used for objective well-being. A close examination of the response rates and missing items within our dataset revealed that these types of questionnaires are not only complicated to fill in as a proxy but also might not capture the small nuances and differences that individuals with PIMD may experience from applying SCC. Both questionnaires measured several aspects of quality of life. It might have been better to focus on the stress or tension that individuals experience, directly related to the IM changes. In addition, using sensor technology to detect tension might provide more objective insights into the experiences of people with PIMD than questionnaires. In future research, measurement of quality of life specifically impacted by care technology should be carefully considered. Rather than administering standardized questionnaires on quality of life, a more qualitative approach, zooming in on the specific changes from RCC to SCC, might be more appropriate [38].

This study was affected by the COVID-19 pandemic in several ways. The start of the data collection was postponed to September 2021, because until then, care organizations faced restrictions with organizing live meetings, and caregivers had to work with additional protective equipment and faced additional work pressure due to the pandemic. Still, COVID-19 outbreaks occurred in some locations during the study period, which altered how continence care was provided, or the way implementation support could be given (remote vs on site). For example, one location reported that participants had to stay in their rooms and all care, including continence care, had to be scheduled, making personalized care difficult. In addition, day activity centers were closed, and in some cases, day activities were organized at residences. When specific locations were in lockdown, data collection was postponed until the end of the lockdown. This occurred at 2 locations during the data collection period. Once out of lockdown, these locations received extra attention from our team to optimize implementation and resolve any remaining issues. The possible impact of the COVID-19 pandemic on the number of leakages, IM changes, and the quality of life of the participants is unknown. However, because COVID-19–related issues were experienced by organizations in both study groups, we do not expect that this had influenced the outcomes of this study.

Finally, an additional limitation of the study may be that although the project leaders of all organizations received extensive guidance in the implementation process [20], issues that might negatively affect SCC implementation could not always be resolved promptly. The challenges included ambassadors leaving the organization, technical issues affecting trust in the technology, and caregivers’ willingness to change their working routines. Individuals with PIMD sometimes have physical deformation, which makes it difficult for the IM to fit well around their bodies. In summary, this study was conducted in a real-life practice, where implementation challenges exist. Although these challenges may have affected SCC implementation, as previously stated, the findings of this study are probably more generalizable and representative of real-world conditions than if the study had been conducted under perfectly controlled circumstances [39].

### Conclusions

This cluster randomized trial revealed that SCC is not effective in reducing the number of leakages but is effective in reducing the number of IM changes for individuals with PIMD living in Dutch residential care facilities. Although statistical analyses showed no effect on the quality of life of individuals with PIMD, we observed that SCC can provide additional value to the participants and their care teams. With additional information generated by the SCC system, work routines can be changed to provide more personalized continence care. Additional research is required to identify the settings in which the implementation of SCC is most meaningful. There needs to be a match between the SCC technology, participants, care teams involved, and support provided.

## Appendix

Multimedia Appendix 1 CONSORT (Consolidated Standards of Reporting Trials) 2010 checklist.

Multimedia Appendix 2 CONSORT-EHEALTH (Consolidated Standards of Reporting Trials of Electronic and Mobile Health Applications and Online Telehealth) V1.6 checklist.

## Abbreviations

CONSORT: Consolidated Standards of Reporting Trials
GLMM: generalized linear mixed model
IM: incontinence material
MIPQ: Mood, Interest and Pleasure Questionnaire
PIMD: profound intellectual and multiple disabilities
QOL-PMD: Quality of Life of Persons with Profound Multiple Disabilities
RCC: regular continence care
SCC: smart continence care

## Data Availability

The datasets generated and analyzed during this study are not publicly available because of the vulnerable nature of the participants, and informed consent was limited to the purpose of this study. A reasonable request can be submitted to the corresponding author.
