# Supplementary material for: Effectiveness of Smart Continence Care for People With Profound Intellectual and Multiple Disabilities: Cluster Randomized Trial
Source: J Med Internet Res. 2025 Jul 31;27:e66389. doi: 10.2196/66389 (PMC12355147; doi:10.2196/66389)
Supplement: Multimedia Appendix 1 [file jmir_v27i1e66389_app1.pdf]

CONSORT 2010 checklist of information to include when reporting a randomised trial*

AND 'Extension to cluster designs'

Section/Topic	Item No	Checklist item	Reported on section
**Title and abstract**			
	1a	Identification as a randomised trial in the title	Title
		Identification as a cluster randomised trial in the title	Title
	1b	Structured summary of trial design, methods, results, and conclusions (for specific guidance see CONSORT for abstracts)	Abstract
		See table 2	
**Introduction**			
Background and objectives	2a	Scientific background and explanation of rationale	Introduction
		Rationale for using a cluster design	Introduction
	2b	Specific objectives or hypotheses	Introduction
		Whether objectives pertain to the cluster level, the individual participant level, or both	Introduction and Methods/Study design
**Methods**			
Trial design	3a	Description of trial design (such as parallel, factorial) including allocation ratio	Methods/Study design
		Definition of cluster and description of how the design features apply to the clusters	Methods/Study design
	3b	Important changes to methods after trial commencement (such as eligibility criteria), with reasons	Methods/Study design
Participants	4a	Eligibility criteria for participants	Methods/Procedure, participant selection and eligibility criteria

Section/Topic	Item No	Checklist item	Reported on page No
		Eligibility criteria for clusters	Methods/Study design
	4b	Settings and locations where the data were collected	Methods/Study design
Interventions	5	The interventions for each group with sufficient details to allow replication, including how and when they were actually administered	Methods/regular continence care and Intervention: smart continence care
		Whether interventions pertain to the cluster level, the individual participant level, or both	See above
Outcomes	6a	Completely defined pre-specified primary and secondary outcome measures, including how and when they were assessed<br>Whether outcome measures pertain to the cluster level, the individual participant level, or both	Table 1<br><br>Methods/Outcome measures
	6b	Any changes to trial outcomes after the trial commenced, with reasons	Change in analyses: Methods/Analyses
Sample size	7a	How sample size was determined<br>Method of calculation, number of clusters(s) (and whether equal or unequal cluster sizes are assumed), cluster size, a coefficient of intracluster correlation (ICC or k), and an indication of its uncertainty	Methods/Sample size<br>See protocol paper
	7b	When applicable, explanation of any interim analyses and stopping guidelines	NA
Randomisation:			
Sequence generation	8a	Method used to generate the random allocation sequence	Methods/Study design
	8b	Type of randomisation; details of any restriction (such as blocking and block size)<br><br>Details of stratification or matching if used	Methods/Study design<br>NA
Allocation concealment mechanism	9	Mechanism used to implement the random allocation sequence (such as sequentially numbered containers), describing any steps taken to conceal the sequence until interventions were assigned<br>Specification that allocation was based on clusters rather than individuals and whether allocation concealment (if any) was at the cluster level, the individual participant level, or both	Methods/Study design

Implementation	10	a) Who generated the random allocation sequence, who enrolled clusters, and who assigned clusters to Interventions<br>b) Mechanism by which individual participants were included in clusters for the purposes of the trial (such as complete enumeration, random sampling)	Methods/Study design<br>Methods/Procedure, participant selection and eligibility criteria
		c) from whom consent was sought (representatives of cluster, or individual participants, or both), and whether consent was sought before or after randomisation	Methods/Ethical considerations
Blinding	11a	If done, who was blinded after assignment to interventions (for example, participants, care providers, those assessing outcomes) and how	NA, statistician: Methods/Study Design
	11b	If relevant, description of the similarity of interventions	NA
Statistical methods	12a	Statistical methods used to compare groups for primary and secondary outcomes<br>How clustering was taken into account	Methods/Analysis<br>Methods/Analysis
	12b	Methods for additional analyses, such as subgroup analyses and adjusted analyses	Methods/Analysis
**Results**			
Participant flow (a diagram is strongly recommended)	13a	For each group, the numbers of participants who were randomly assigned, received intended treatment, and were analysed for the primary outcome<br>For each group, the numbers of clusters that were randomly assigned, received intended treatment, and were analysed for the primary outcome	Figure 2<br>Figure 2
	13b	For each group, losses and exclusions after randomisation, together with reasons<br>For each group, losses and exclusions for both clusters and individual cluster members	Figure 1<br>Figure 1
Recruitment	14a	Dates defining the periods of recruitment and follow-up	Page 2
	14b	Why the trial ended or was stopped	Sample size reached
Baseline data	15	A table showing baseline demographic and clinical characteristics for each group<br>Baseline characteristics for the individual and cluster levels as applicable for each group	Table 2 and 3<br>NA, analyses on individual level
Numbers analysed	16	For each group, number of participants (denominator) included in each analysis and whether the analysis was by original assigned groups<br>For each group, number of clusters included in each analysis	Table 2, 3, 4<br>Figure 2

Outcomes and estimation	17a	For each primary and secondary outcome, results for each group, and the estimated effect size and its precision (such as 95% confidence interval)	See table 5
		Results at the individual or cluster level as applicable and a coefficient of intracluster correlation (ICC or k) for each primary outcome	NA
	17b	For binary outcomes, presentation of both absolute and relative effect sizes is recommended	Table 4 and 5
Ancillary analyses	18	Results of any other analyses performed, including subgroup analyses and adjusted analyses, distinguishing pre-specified from exploratory	Results/Per protocol and completer analyses, and, Exploratory subgroup analysis
Harms	19	All important harms or unintended effects in each group (for specific guidance see CONSORT for harms)	Results/Per protocol and completer analyses
**Discussion**			
Limitations	20	Trial limitations, addressing sources of potential bias, imprecision, and, if relevant, multiplicity of analyses	Discussion/Strengths and limitations
Generalisability	21	Generalisability (external validity, applicability) of the trial findings	Discussion
		Generalisability to clusters and/or individual participants (as relevant)	
Interpretation	22	Interpretation consistent with results, balancing benefits and harms, and considering other relevant evidence	Discussion
**Other information**			
Registration	23	Registration number and name of trial registry	Available
Protocol	24	Where the full trial protocol can be accessed, if available	Reference 13
Funding	25	Sources of funding and other support (such as supply of drugs), role of funders	Available

*We strongly recommend reading this statement in conjunction with the CONSORT 2010 Explanation and Elaboration for important clarifications on all the items. If relevant, we also recommend reading CONSORT extensions for cluster randomised trials, non-inferiority and equivalence trials, non-pharmacological treatments, herbal interventions, and pragmatic trials. Additional extensions are forthcoming: for those and for up to date references relevant to this checklist, see www.consort-statement.org.
